# Main Processed Hypoallergenic Foods: A Potential Tool to Improve Informed Dietary Choices in Children with IgE-Mediated Food Allergies

**DOI:** 10.3390/children12070915

**Published:** 2025-07-11

**Authors:** Luca Pecoraro, Simona Barni, Francesca Mori, Mattia Giovannini, Riccardo Castagnoli, Stefania Arasi, Carla Mastrorilli, Francesca Saretta, Lucia Liotti, Lucia Caminiti, Angela Klain, Mariannita Gelsomino, Michele Miraglia Del Giudice, Gian Luigi Marseglia, Elio Novembre

**Affiliations:** 1 Pediatric Unit, Ospedale Vito Fazzi, ASL Lecce, 73100 Lecce, Italy; 2 Allergy Unit, Meyer Children’s Hospital IRCCS, 50139 Florence, Italyfrancesca.mori@meyer.it (F.M.);; 3 Department of Health Sciences, University of Florence, 50139 Florence, Italy; 4 Department of Clinical, Surgical, Diagnostic and Pediatric Sciences, University of Pavia, 27100 Pavia, Italy; 5 Pediatric Clinic, Fondazione IRCCS Policlinico San Matteo, 27100 Pavia, Italy; 6 Translational Research in Pediatric Specialties Area, Division of Allergy, Bambino Gesù Children’s Hospital [IRCCS], 00165 Rome, Italy; 7 Pediatric and Emergency Department, Pediatric Hospital Giovanni XXIII, AOU Policlinic of Bari, 70126 Bari, Italy; 8 Pediatric Department, Latisana-Palmanova Hospital, Azienda Sanitaria Universitaria Friuli Centrale, 33100 Udine, Italy; francesca.saretta@asufc.sanita.fvg.it; 9 Pediatric Unit, Department of Mother and Child Health, Salesi Children’s Hospital, 60123 Ancona, Italy; 10 Pediatric Unit, Department of Human Pathology in Adult and Development Age “Gaetano Barresi”, Allergy Unit, AOU Policlinico Gaetano Martino, 98124 Messina, Italy; 11 Department of Woman, Child and of General and Specialized Surgery, University of Campania ‘Luigi Vanvitelli’, 80138 Naples, Italy; angela.klain@studenti.unicampania.it (A.K.);; 12 Allergy Unit, Pediatrics Area, Department of Woman and Child Health, Policlinico Gemelli University Foundation IRCCS, Catholic University of Sacre Hearth, 00168 Rome, Italy; mariannita.gelsomino@guest.policlinicogemelli.it

**Keywords:** processed foods, processing method, big 9, oral allergen immunotherapy, food allergy, allergenic foods, processed forms of food allergens

## Abstract

In the context of IgE-mediated food allergies in children, the use of hypoallergenic foods may offer an appropriate solution for enabling informed dietary choices and reducing reactivity to allergenic foods. It is well established that certain foods can alter their allergenicity depending on the method of processing. As such, processed foods may serve both as an alternative dietary option and as a useful tool in oral immunotherapy for children with IgE-mediated food allergies. Nevertheless, an oral food challenge is always recommended when a pediatric allergist considers incorporating processed foods into a child’s diet. This review aims to explore the potential use of processed forms of the nine major food allergens in IgE-mediated food allergies, supporting pediatric allergists in partially liberalizing children’s diets and facilitating the development of tolerance.

## 1. Introduction

More than 20 years ago, Ortolani et al. [1] proposed that food allergies could be addressed through three primary strategies: improving food labeling, avoiding culprit foods, and developing hypoallergenic foods using food technology. Over time, the combination of allergen avoidance and the implementation of appropriate action plans for allergic reactions has become a cornerstone in the management of food allergies [2]. Concurrently, oral immunotherapy has emerged as an innovative treatment for children with persistent, severe allergies to cow’s milk, hen’s eggs, or peanuts [2]. In this context, the use of omalizumab, a monoclonal anti-IgE antibody, shows promise in both children and adults with multiple food allergies, as it increases the reaction threshold to common allergens [3]. However, the latest GA2LEN guidelines have highlighted significant gaps in the evidence regarding the safety and efficacy of these various strategies [2]. Notably, the guidelines did not explore in depth the potential of developing hypoallergenic foods through food technology [2]. Hypoallergenic foods may provide a useful strategy for making informed dietary choices and reducing reactivity to allergenic foods. It is well known that structural changes occurring during food processing can either diminish or enhance the allergenicity of food proteins by altering the exposure of three-dimensional allergenic epitopes, modifying protein conformation, or forming new protein complexes [4]. Understanding processing-induced changes is essential for evaluating allergenic risk and establishing tolerance thresholds in individuals with food allergies [5,6]. The allergist plays a key role in identifying major allergens, which is a prerequisite for the potential development of hypoallergenic foods via food technology [1,7,8,9]. This review explores the potential role of processed hypoallergenic foods in supporting informed dietary choices and promoting the development of tolerance in children with IgE-mediated food allergies.

## 2. Research Strategies and Literature Analysis

We conducted a non-systematic review of the most relevant studies on “processed hypoallergenic food” using the PubMed and Cochrane Library databases, covering publications from January 1932 to November 2024. Eligible manuscripts included randomized controlled trials, case reports, reviews, systematic reviews, cohort studies, case-control studies, and observational studies. Articles not published in English were excluded. The search terms used included the following: “processed hypoallergenic food”, “processing food”, “processing hypoallergenic cow’s milk allergy”, “processing hypoallergenic egg allergy”, “processing hypoallergenic soy allergy”, “processing hypoallergenic fish allergy”, “processing hypoallergenic shellfish allergy”, “processing hypoallergenic wheat allergy”, “processing hypoallergenic peanut allergy”, “processing hypoallergenic tree nut allergy”, “processing hypoallergenic sesame allergy”, and “processing oral allergen immunotherapy”.

## 3. Food Processing and Modification of Allergenicity

Food processing involves transforming raw materials into final products suitable for human consumption. Historically, the modification of food allergenicity was discovered incidentally [10,11]. Traditional food processing techniques, such as cooking, curing, and smoking, were primarily developed to extend shelf life and facilitate food transport. Pasteurization and other heat treatments were later introduced to enhance food safety by reducing spoilage and eliminating pathogenic microorganisms [10,12]. More recently, novel technologies have aimed to improve both the palatability and productivity of food products [10,12]. Currently, food industry processing methods are broadly classified into thermal and non-thermal techniques [13]. Thermal methods include baking, steaming, boiling, roasting, pasteurization, ultra-high-temperature (UHT) treatment, drying, smoking, and Maillard reaction/glycation processes [12,13]. Non-thermal technologies comprise high-pressure processing, microwave treatment, cold or atmospheric plasma, freezing, ultrasound, irradiation/UV, pulsed electric fields, membrane processing, enzymatic treatments (hydrolysis, lipolysis), and fermentation [13,14]. These approaches may also be combined, as in autoclaving or evaporation processes [13,15]. The consumption of processed foods has increased significantly in recent years [16]. This shift has altered the characteristics of food products consumed by both children and adults, highlighting the need for allergists to guide informed dietary choices in patients with food allergies [10]. Interest in this topic has grown due to evidence that processing can modify the allergenicity of certain foods [10]. In particular, changes in protein function, stability, and glycosylation induced by processing may influence their allergenic potential [4,17]. The surrounding food matrix may also play a role in this modulation [18]. Processing techniques implicated in altering allergenic properties include heating, fermentation, endogenous enzymatic hydrolysis, enzymatic and acid hydrolysis, high-pressure processing, pH alteration, or combinations of these methods [4,19,20]. From a pathophysiological perspective, it is hypothesized that processing affects allergen susceptibility to digestive enzymes, either by altering protein structures or degrading the allergenic proteins entirely [13]. Considering that adaptive immune responses are antigen-specific and depend on the recognition of particular antigens during presentation [21] and that this mechanism is disrupted in IgE-mediated food allergies [22], processing may also affect antigen–antibody interactions [13]. Tolerance development involves several immunological components, including regulatory T cells, antigen-presenting cells, TGF-β, IL-10, and IgG4 antibodies [5]. From a practical standpoint, although most food allergens are proteins, not all dietary proteins are allergenic [23]. A limited number of foods are primarily responsible for IgE-mediated allergic reactions. These include cow’s milk (5.7%), hen’s eggs (2.4%), wheat (1.6%), peanuts (1.5%), fish (1.4%), tree nuts (0.9%), soy (0.5%), and shellfish (0.4%) [24], collectively identified by the U.S. Food and Drug Administration as the nine major food allergens (commonly referred to as the “Big 9”) [25].

## 4. Cow’s Milk

When breastfeeding is not possible, infants are typically given infant formula [26,27]. As a result, cow’s milk allergy is often the earliest food allergy to develop in children [28]. Cow’s milk contains two main groups of proteins: the insoluble casein fraction (80%) and the soluble whey proteins (20%) [26]. Allergic reactions may involve either or both of these protein groups [28]. In general, raw milk is not consumed directly [4]. It is processed before commercialization, and various processing methods are known to alter the stability of milk proteins [13]. These include pasteurization, sterilization, denaturation, non-enzymatic glycation, UHT (ultra-high-temperature) processing, vacuum condensing, spray drying, and irradiation [4]. Preliminary evidence suggests that pasteurization may increase milk allergenicity by promoting protein activation, aggregation, and enhanced binding to mast cells. In contrast, sterilization appears to reduce the IgE response. Denaturation and non-enzymatic glycation destroy epitopes, rendering them inaccessible. Meanwhile, UHT processing, vacuum condensing, spray drying, and irradiation do not significantly affect milk allergenicity [4]. Recent hypotheses suggest that UHT homogenized milk may promote the formation of “lipid–protein nanoparticle” structures, which can expose cryptic antigenic sites and trigger pathological immune responses, such as eosinophilic esophagitis [29]. Boiling is another form of home processing. Boiling milk for 10 min reduces its allergenicity by inactivating β-lactoglobulin and albumin, while casein remains stable and still yields a positive skin prick test (SPT) [30]. Casein retains its allergenicity even with longer boiling durations [13]. Although heat treatment reduces the overall allergenicity, subsequent gastric digestion may increase the immunogenicity of cow’s milk allergens, requiring additional modification [31]. While heating does not eliminate the IgE-binding capacity of lactoglobulin, it enhances its digestibility, which may explain its reduced allergenicity [32]. Fermentation is another method of processing cow’s milk, as seen in yogurt production. Fermentation reduces milk allergenicity, either by introducing probiotics that modulate the Th1/Th2 balance or through the activity of proteases that degrade allergenic epitopes [31]. Another form of processed milk—fully matured cheese—also shows reduced allergenicity. Evidence suggests that 58–78% of children with cow’s milk allergy can tolerate fully matured cheeses, possibly due to the partial digestion of casein during the maturation process, which further facilitates allergen degradation during digestion [33,34]. Baked milk is another processed form with reduced allergenic potential. Dry heat induces structural changes in milk proteins through both heat exposure and glycation, which can enhance digestibility and reduce immunoreactivity [13,35]. In practice, baked milk is often introduced in the form of muffins. This specific cooking method generates complex milk–food interactions that may modulate immunoreactivity [36]. Compared to milk baked at 180 °C for 10 min, muffins have been shown to induce greater reductions in allergenicity, as demonstrated by in vitro tests [36]. Baked milk has also been associated with increased tolerance to liquid cow’s milk and other dairy products [37]. However, the evidence on allergenicity reduction through milk processing remains conflicting. Teodorowicz et al. [38] demonstrated that both the heating and glycation of beta-lactoglobulin can enhance its immunogenicity, with glycation showing a more pronounced effect than heating alone. Similarly, Abbring et al. [39] found that raw cow’s milk had lower in vitro allergenicity compared to processed milk. The recent increase in eosinophilic esophagitis has led to the proposal of a “Processed Milk Hypothesis”. This posits that UHT treatments (135 °C) and homogenization result in the formation of small fat droplets carrying milk proteins, which then become targets for circulating IgG4 and may trigger IgG4-mediated immune responses [29,40]. Cow’s milk must be digested after ingestion, and the stability of milk proteins during digestion varies depending on the processing method [13]. Pasteurized and UHT-treated caseins are generally digested rapidly in the stomach, although caseins in UHT milk show greater resistance to gastric digestion. Thus, processing methods must be considered when assessing the stability and allergenicity of milk proteins. In the context of oral immunotherapy, baked milk may play a useful role in promoting tolerance development [34].

## 5. Hen’s Eggs

Hen’s eggs are consumed globally and are commonly found in a wide range of food products. These can be broadly categorized as either raw/less heated or baked/extensively heated. Raw or lightly cooked hen’s eggs are typically present in dishes such as French toast, custard, quiche, fresh mayonnaise, and Caesar salad dressing. Extensively heated or baked forms include cakes, waffles, muffins, pancakes, egg noodles, egg pasta, and bread [41]. From an allergenic perspective, distinguishing between less- and more-heated forms of hen’s eggs is critical [13]. The allergenic potential of hen’s egg proteins can be significantly altered by cooking or processing. Egg white contains proteins with considerably higher allergenic potential compared to egg yolk. The four major allergens in egg white are ovalbumin, ovotransferrin, ovomucoid, and lysozyme. In the yolk, α-livetin and lipoprotein YGP42 are the main allergenic proteins [32]. Among these, ovalbumin and ovomucoid are considered the most clinically relevant egg allergens [13]. Chemically, ovomucoid is highly resistant to both proteolytic digestion and heat denaturation, whereas ovalbumin is more heat sensitive [42]. For example, heating ovomucoid for 3 min at 100 °C does not significantly reduce its allergenicity, whereas the same treatment reduces the allergenicity of ovalbumin by 90% [43]. When followed by gastric digestion, hen’s egg allergens may undergo further structural changes. Egg proteins are digested by various proteases in the stomach [44]. Unlike ovomucoid, ovalbumin shows a marked reduction in sensitizing potential and allergic symptoms after digestion [13]. In vivo studies have confirmed that heat treatment reduces the allergenicity of ovalbumin but not ovomucoid. More than 50% of children with hen’s egg allergy can tolerate cooked eggs, and an even higher percentage can tolerate baked eggs. This increased tolerance is partly attributed to the presence of wheat flour, which forms disulfide bonds with ovomucoid during baking, followed by precipitation, reducing its allergenic potential [45]. Other cooking methods—such as steaming, frying, and baking—also reduce egg allergenicity, with frying showing particularly notable effects in in vitro studies [46]. More recently, processing methods involving the addition of phenolic compounds have been explored; however, no method has been found to completely eliminate the allergenic potential of egg proteins [47]. Among all processing methods, baking has shown the most promise in oral immunotherapy. Regular consumption of baked hen’s eggs has been shown to accelerate the development of clinical tolerance [48]. De Viliger et al. [49] proposed a gradual, short-arm protocol to help children who tolerate baked egg progress safely toward the tolerance of raw egg at home.

## 6. Soy

Soy is commonly found in foods such as tofu (soybean curd), edamame (whole soybeans), tempeh (cooked and fermented soy), miso (fermented soybean paste), soy milk, soy flour, and soy sauce [50]. Soybean seeds contain approximately 37% protein, with the most relevant allergenic proteins being PR-10 protein (Gly m 4), β-conglycinin (Gly m 5), glycinin (Gly m 6), and 2S-albumin (Gly m 8) [50]. Among these, Gly m 5 and Gly m 8 are primarily involved in the initial sensitization to soy. Gly m 8, in particular, has shown high diagnostic value in children with soy allergy [51]. Gly m 4, on the other hand, plays a key role in clinical cross-reactivity with Bet v 1, the major birch pollen allergen [52]. Soy-based food products undergo various processing steps, during which the structure and properties of soy proteins may be altered, leading to either reduced or enhanced allergenicity [4]. Among the processing methods, fermentation is the most effective at reducing soybean allergenicity. This is because it destroys allergenic epitopes and produces metabolites that can decrease the immune system’s sensitivity to soy. The most well-known fermented soy product is soy sauce, which is generally well tolerated by individuals with soy allergies [53,54,55]. No other processing method has been shown to completely eliminate soy allergenicity [4]. To date, no studies have evaluated the use of processed soy in oral immunotherapy.

## 7. Fish

The primary fish allergen is parvalbumin, a calcium-binding protein found in fish muscle [4]. Other notable fish allergens include aldolases, enolases, collagen, and tropomyosin [56]. The concentration of parvalbumin varies across different fish species, [57] resulting in species-specific differences in allergenicity [58]. Parvalbumin is predominantly located in the white muscle of fish. In contrast, species such as swordfish and tuna, which contain more red muscle, have lower parvalbumin content. Parvalbumin is generally resistant to both processing and digestion [59,60,61]. As a result, its allergenicity can depend on multiple factors, including fish species, digestive conditions, the specific processing method, and the food matrix [13]. Processing techniques such as heating, boiling, steaming, and freezing have been shown to reduce the allergenicity of parvalbumin to varying degrees [13]. However, findings regarding the effect of irradiation on parvalbumin allergenicity are inconsistent [13,31]. Fish can also be processed into seafood substitutes. For example, surimi and canned tuna have been shown to reduce fish allergenicity in vitro and may be tolerated by some individuals with fish allergies [61,62]. Pecoraro et al. [63] demonstrated that canned tuna was tolerated in vivo by children with IgE-mediated fish allergy. Given that approximately 30% of fish products are consumed in canned form in developed countries [57], this has important nutritional implications. Fish is an essential dietary source of vitamins A, B, and D, as well as omega-3 fatty acids [64]. In the context of allergen-specific immunotherapy, preliminary evidence indicates that recombinant hypoallergenic carp parvalbumin may induce tolerance in vitro in individuals with fish allergy [65].

## 8. Shellfish

Research on the allergenicity of processed shellfish remains limited. The primary shellfish allergen is tropomyosin [66,67]. Other identified allergens include arginine kinase, myosin light chain, troponin C, and sarcoplasmic calcium-binding protein [68,69]. Certain processing techniques—such as heat combined with reverse-pressure sterilization or microwaving—may reduce shellfish allergenicity [69,70]. However, heat processing alone does not significantly reduce the allergenicity of tropomyosin [71]. Specifically, Nagpal et al. [72] demonstrated that the anaphylaxis-inducing activity of shrimp remained unchanged after cooking at 100 °C [72]. To date, no in vivo studies have confirmed tolerance to processed shellfish [73]. Nonetheless, in the field of allergen-specific immunotherapy, early evidence suggests that a recombinant hypoallergenic variant of Cra g 1 can reduce the allergenic potency of oyster tropomyosin in vitro [74]. Additionally, hypoallergenic derivatives of *Scylla* paramyosin (mud crab) show promise as candidates for allergen immunotherapy targeting crab allergies [75].

## 9. Wheat

Wheat is one of the most widely consumed cereals globally and is found in a broad range of products, including bread, pasta, noodles, breakfast cereals, baked goods, sauces, and ice cream [76]. The main allergenic proteins in wheat are albumins, globulins, gliadins, and glutenins [77]. Gliadins and glutenins are classified as the storage proteins of wheat [77]. Heating wheat at high temperatures, especially in the presence of a carbohydrate matrix (as in pasta), renders wheat allergens more resistant to digestion [4]. Certain processing methods, such as acid hydrolysis, fermentation, and irradiation, have been reported to reduce the allergenicity of specific wheat proteins; however, the evidence remains inconsistent [31]. In contrast, it has been demonstrated that deamidation of wheat gliadin significantly reduces its allergenicity [78]. Additionally, processing wheat proteins with thioredoxin has been shown to lower the allergenicity of both gliadin and glutenin [79]. Based on this evidence, in vitro studies suggest that specially modified wheat products—such as ω-5 gliadin-free wheat flour, deamidated noodles, and “O-free” wheat flour—may serve as examples of hypoallergenic processed foods for individuals with wheat allergy [80,81,82]. However, no in vivo studies to date have confirmed the development of tolerance to processed wheat. Notably, a case report described a boy with a wheat allergy who experienced an allergic reaction after ingesting only a few micrograms of wheat in a processed form, specifically from packaged rice crackers [83]. In the context of allergen-specific immunotherapy, wheat oral immunotherapy has shown promise as a safe and effective treatment for children with wheat allergy [84]. Moreover, an in vitro-developed pepsin-digested gliadin exhibited hypoallergenic properties and the potential to induce immune tolerance [84].

## 10. Peanut

Peanut allergy is a global concern [4]. Notably, peanut processing methods vary across countries, which may help explain the wide geographical differences in peanut allergy prevalence [4,13]. For example, peanut allergy is less common in countries where peanuts are typically boiled, as opposed to countries where they are mainly consumed roasted. From an allergenic perspective, 17 distinct peanut allergens have been identified (Ara h 1 to Ara h 17), each with different biochemical properties. Among the most clinically relevant: Ara h 2, 6, and 7 are 2S albumins; Ara h 1 and 3 belong to the cupin family; Ara h 9, 16, and 17 are non-specific lipid transfer proteins (nsLTPs); Ara h 5 is a profilin; and Ara h 8 is a PR-10 protein [85]. Ara h 2 and Ara h 6 are particularly notable for their stability during digestion [86]. In vitro studies have shown that boiling peanuts reduces their allergenicity by denaturing allergenic proteins and leaching low-molecular-weight proteins into the cooking water. In contrast, roasting peanuts increases their allergenicity by generating advanced glycation end-products (AGEs). Simple thermal processing does not significantly alter peanut allergenicity on its own [74]. However, when thermal processing is combined with autoclaving, a significant reduction in allergenicity is observed [87]. Baked peanuts, on the other hand, show no notable change in allergenicity [87]. Other processing methods—such as irradiation, fermentation, and protease hydrolysis—have also been found to reduce peanut allergenicity [31,88,89]. In vivo studies have further explored the allergenic potential of specific peanut products, including crude and refined peanut oil, as well as boiled peanuts. Hourihane et al. [90] found that approximately 10% of individuals with IgE-mediated peanut allergy reacted to crude peanut oil. In contrast, no reactions were observed in subjects exposed to refined (raffinate) peanut oil [89]. This difference is attributed to the chemical and physical purification processes used in refining, which remove allergenic proteins [90,91]. Indeed, no detectable protein is found in refined peanut oil [91]. Crude peanut oil, by contrast, contains significant amounts of residual protein and is rarely used, except when a distinct peanut flavor is desired, thus posing a risk for allergic reactions in sensitized individuals. Refined peanut oil, in comparison, is generally considered safe for individuals with peanut allergies [90]. Another in vivo study by Turner et al. [92] demonstrated that boiled peanuts were well tolerated by four children with confirmed peanut allergy. This tolerance may be explained by the reduction of allergenic proteins—particularly Ara h 2, 6, and 7—during the boiling process [93]. Immormino et al. [94] also showed that mice fed peanut butter developed tolerance to peanuts when used as part of an immunotherapy protocol involving processed peanuts. Currently, peanut oral immunotherapy is available and has proven effective in both children and adults [95,96,97].

## 11. Tree Nuts

The global consumption of tree nuts and seeds is increasing [4,98,99]. The most clinically significant tree nut allergens include pecan (Car i), almond (Pru du), pistachio (Pis v), walnut (Jug r), hazelnut (Cor a), Brazil nut (Ber e), and cashew nut (Ana o) [4]. Tree nut allergy may develop through direct sensitization to major allergenic proteins—such as lipid transfer proteins (LTPs), 2S albumins, and seed storage proteins—or through cross-reactivity between Bet v 1 (a major birch pollen allergen) and PR-10 proteins [4,100,101]. Processing methods can influence the allergenicity of these proteins [4]. Heat treatment, for example, alters the structure of PR-10 proteins and profilins, thereby reducing their allergenicity. However, heat has a minimal effect on the allergenicity of LTPs and seed storage proteins due to their structural stability. In vivo studies by Hansen et al. and Worm et al. demonstrated that 100% of patients with birch pollen-related hazelnut allergy experienced symptoms after consuming raw hazelnuts. However, when given roasted hazelnuts, 65% reported no allergic symptoms, and the remaining 35% experienced only mild symptoms, indicating a direct relationship between thermal processing and reduced allergenicity [102,103]. In vitro studies on tree nut processing remain limited. Cabanillas et al. showed that sonication can reduce the allergenicity of pistachio proteins [104]. However, due to the resistance of seed storage proteins and LTPs to denaturation and proteolysis, no studies have demonstrated a consistent reduction in allergenicity for other specific tree nuts via food processing. Tree nuts are generally considered safe candidates for allergen-specific immunotherapy [105]. Additionally, the use of processed tree nuts is being explored; for example, the irradiation of tree nut flours has been tested and shown to be safe [106].

## 12. Sesame

Sesame is cultivated in tropical and subtropical regions and has seen increased global use in recent years. It is commonly found in bakery products (e.g., breads, cookies, bagels, and chocolate), condiments (e.g., shichimi, za’atar, gomasio, and togarashi), and confectionery. Sesame is also used in cosmetics [98]. It has recently been classified as a “new major allergen” [98]. At the molecular level, sesame allergy may result from sensitization to several key proteins, including 2S albumins (Ses i 1 and Ses i 2), 7S vicilin-like globulin (Ses i 3), oleosins (Ses i 4 and Ses i 5), and 11S globulins (Ses i 6 and Ses i 7) [98]. These proteins are also present in other allergenic foods, which can lead to cross-reactivity—particularly with tree nuts—in clinical settings [107]. As with other food allergens, both thermal and non-thermal processing can affect the allergenicity of sesame. However, in vitro studies have shown that heating and boiling do not significantly alter sesame’s allergenic potential [98]. The effects of roasting, microwaving, irradiation, and enzymatic hydrolysis remain inconclusive [98]. In contrast, high-pressure processing, fermentation, and glycation have been shown to reduce sesame allergenicity [98]. Interestingly, many individuals sensitized to sesame react to tahini (a sesame paste) but not to whole seeds. This is likely due to the higher protein content in tahini compared to intact seeds [108,109]. As a result, children with sesame allergies may have variable thresholds, tolerating some forms of sesame (e.g., whole seeds, sesame oil) while reacting to others (e.g., crushed seeds, flour, paste) [109]. Regarding immunotherapy, promising results have been achieved using processed forms of sesame. Nachshon et al. reported that oral immunotherapy (OIT) with raw tahini was effective and safe, with 88.4% of treated pediatric and adult patients achieving desensitization [109]. Similarly, studies by Zielinska and Chua also demonstrated the efficacy and safety of OIT using tahini [110,111]. Finally, a study by Kaman et al. [112] found that a specific natural variety—black sesame—may be a suitable candidate for oral immunotherapy due to its lower allergenic potential.

## 13. The Children’s Perspective

Specific processing methods applied to food allergens can offer alternative dietary options for individuals with IgE-mediated food allergies. These processed forms include boiled milk, yogurt, baked milk, cooked hen’s eggs, baked hen’s eggs, soy sauce, surimi, canned fish, "ω-5 gliadin-free" wheat flour, “O-free” wheat flour, refined (raffinate) peanut oil, boiled peanuts, and roasted hazelnuts (Table 1).

Protein allergenicity is determined by the presence of specific epitopes that are recognized by immunoglobulins, thereby triggering an allergic response. Processing can modify these epitopes, potentially reducing the allergenicity of the food and making it more tolerable [113]. Thermal processing methods are the most common ones [114], although non-thermal methods may also play a role [13]. Risk management in food allergies remains a challenge for clinicians, especially pediatric allergists [115]. While evidence-based medicine relies on the strength of scientific evidence, the current low level of in vivo studies assessing tolerance to processed foods in children with food allergies limits the ability to introduce these foods into the diet without prior testing via an oral food challenge in a hospital setting [115,116]. Koosakulchai et al. [117] demonstrated that children who successfully passed an oral food challenge with a medium dose of cow’s milk were subsequently able to consume processed dairy products at home. Similarly, Miceli Sopo et al. [34] found that a negative skin prick-by-prick test could be used to identify children who may safely consume processed cow’s milk without undergoing an oral food challenge. Monaco et al. [118] also reported that yogurt could be tolerated by children with IgE-mediated cow’s milk allergy. In the context of hen’s egg allergy, studies have shown that 88% of children can tolerate baked hen’s eggs, and 56% can tolerate cooked hen’s eggs [119]. The same study demonstrated a 100% negative predictive value for prick-by-prick testing, suggesting that a negative result could justify the introduction of these processed egg forms without an oral food challenge. Saifi et al. [120] confirmed these findings specifically for baked eggs. With respect to fish allergies, canned tuna has been shown to be tolerated by children with IgE-mediated fish allergy [121]. For IgE-mediated peanut allergy, studies indicate that children may tolerate boiled peanuts without experiencing allergic reactions [92]. To date, no in vivo studies have confirmed tolerance to processed forms of soy, shellfish, wheat, tree nuts, or sesame in children. However, in the setting of oral immunotherapy, processed foods such as baked milk and baked hen’s eggs may play a valuable role [49,119]. Preliminary evidence also supports the use of tahini as a safe and effective option for children with IgE-mediated sesame allergy [109,110,111]. Still, no in vivo studies currently support the use of processed soy, fish, peanut, shellfish, wheat, or tree nuts in pediatric immunotherapy.

## 14. Conclusions

**Figure 1:**
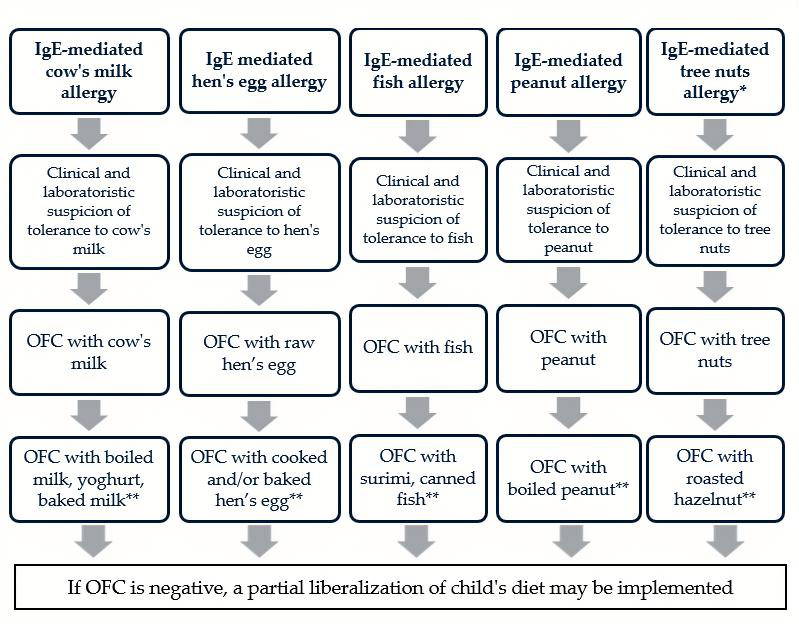
Proposal of algorithm involving processed allergens in the assessment of tolerance to food allergens in pediatric age. * in case of cosensitization to birch. ** to be performed if OFC with an unprocessed allergen is positive. OFC: oral food challenge.

Processed foods may offer an alternative dietary option for children with IgE-mediated food allergies. Preliminary evidence suggests that specific processing methods can increase tolerability and potentially support their use in oral immunotherapy. However, predicting the sensitization capacity and allergenicity of processed foods in real-life scenarios remains impossible at present. Historically, it has been theorized that certain processing techniques can modify the allergenic potential of foods, but this does not always translate into clear safety recommendations [122]. Some studies have demonstrated that a negative prick-by-prick test to baked milk and hen’s eggs may reliably predict tolerance, supporting the safe introduction of these processed forms into the diets of selected children [34,119]. To date, in vivo studies have shown that processed cow’s milk, egg, fish, peanut, and tree nuts (selected cases) may be tolerated in pediatric age. At the same time, no such studies have confirmed tolerance to processed soy, shellfish, wheat, sesame or tree nuts [92,118,119,120,121]. An algorithm proposal can be suggested for clinical practice (Figure 1).

Still, a proposal of more robust evidence is needed before this approach can be broadly recommended in clinical practice. It is important to note that many allergens remain stable, and current processing methods have not succeeded in effectively desensitizing all allergenic foods. Moreover, some processing techniques—such as those used in UHT homogenized milk—may expose cryptic antigenic sites capable of inducing a pathological immune response [29]. An oral food challenge remains essential when a pediatric allergist is considering the introduction of processed food as an alternative dietary option for a child [122]. In the context of oral immunotherapy, processed foods may present less risk in children than in adults [123], owing to the greater plasticity of the pediatric immune system and its increased responsiveness to immunomodulation [124]. The evidence to date highlights the safety, nutritional value, quality-of-life benefits, and public health relevance of incorporating processed foods into the management of food allergies [10]. In this context, the role of the dietitian is crucial, ensuring individualized dietary assessments and patient-centered counseling based on the specific processed foods deemed tolerable by the pediatric allergist [125,126]. Building on the current evidence and the emerging role of food processing in expanding dietary choices and supporting tolerance induction in children, future research should focus on identifying allergenic proteins in various foods and characterizing how these proteins are altered during processing. This could contribute to the development of safe, high-quality hypoallergenic foods.

## Figures and Tables

**Table 1:** Big 9—foods and related potentially less allergenic processing methods associated with potentially tolerated processing food in real life and induction of tolerance. All processed forms need an oral food challenge before reintroduction.

Big 9—Foods	Potentially Less Allergenic Processing Method	Demonstrated Tolerance to Processed Forms	Potential Use in Oral Immunotherapy
Cow’s milk	Sterilization, denaturation, non-enzymatic glycation, boiling, fermentation, and baking	Boiled milk, yoghurt, and baked milk	Baked milk
Hen’s egg	Boiling, baking, frying, and steaming	Cooked hen’s egg and baked hen’s egg	Baked hen’s eggs
Soy	Fermentation	Soy sauce	This statement is not demonstrated
Fish	Heating, boiling, steaming, and freezing	Surimi, canned fish	This statement is not demonstrated
Shellfish	Heat in combination with reverse-pressure sterilization, microwaving	This statement is not demonstrated	This statement is not demonstrated
Wheat	Acid-hydrolysis, fermentation, irradiation, deamidation of wheat gliadin, and thioredoxin process	"ω-5 gliadin-free" wheat flour, “O-free” wheat flour	This statement is not demonstrated
Peanut	Boiling, boiling in combination with autoclave, irradiation, fermentation, and protease hydrolysis	Raffinate peanut oil and boiled peanut	This statement is not demonstrated
Tree nuts	Boiling and irradiation	Roasted hazelnut (only in patients with cosensitization to birch)	This statement is not demonstrated
Sesame	High-pressure processing and fermentation	This statement is not demonstrated	Tahini

## Data Availability

Not applicable.
